# The Relationship between Postural Stability and Lower-Limb Muscle Activity Using an Entropy-Based Similarity Index

**DOI:** 10.3390/e20050320

**Published:** 2018-04-26

**Authors:** Chien-Chih Wang, Bernard C. Jiang, Pei-Min Huang

**Affiliations:** 1 Department of Industrial Engineering and Management, Ming Chi University of Technology, New Taipei City 243, Taiwan; 2 Department of Industrial Management, National Taiwan University of Science and Technology, Taipei City 106, Taiwan; 3 Department of Industrial Engineering and Management, Yuan Ze University, Chung-Li 320, Taiwan

**Keywords:** experiment of design, empirical mode decomposition, signal analysis, similarity indices, synchronization analysis

## Abstract

The aim of this study is to see if the centre of pressure (COP) measurements on the postural stability can be used to represent the electromyography (EMG) measurement on the activity data of lower limb muscles. If so, the cost-effective COP data measurements can be used to indicate the level of postural stability and lower limb muscle activity. The Hilbert–Huang Transform method was used to analyse the data from the experimental designed to examine the correlation between lower-limb muscles and postural stability. We randomly selected 24 university students to participate in eight scenarios and simultaneously measured their COP and EMG signals during the experiments. The Empirical Mode Decomposition was used to identify the intrinsic-mode functions (IMF) that can distinguish between the COP and EMG at different states. Subsequently, similarity indices and synchronization analyses were used to calculate the correlation between the lower-limb muscle strength and the postural stability. The IMF5 of the COP signals and the IMF6 of the EMG signals were not significantly different and the average frequency was 0.8 Hz, with a range of 0–2 Hz. When the postural stability was poor, the COP and EMG had a high synchronization with index values within the range of 0.010–0.015. With good postural stability, the synchronization indices were between 0.006 and 0.080 and both exhibited low synchronization. The COP signals and the low frequency EMG signals were highly correlated. In conclusion, we demonstrated that the COP may provide enough information on postural stability without the EMG data.

## 1. Introduction

Postural stability is a complex process of coordination of the body that resists gravity and involves multiple coordination activities such as biomechanics, sensations, and mobility. Postural stability control is a key skill that affects motion performance. Static postural stability positively correlates with age from 2 years of age to 12 years of age. However, after reaching middle age, the standing postural stability becomes inversely correlated with age. Maintaining a good postural stability is a key factor for maintaining a good quality of life while ageing. When abnormalities occur in the vision, the vestibular or somatosensory systems, which controls postural stability and muscle endurance, can help for maintaining good postural stability [1]. Porter et al. [2] highlighted that the muscle endurance of the somatosensory system gradually decreases with age. Chodzko-Zajko et al. [3] indicated that resistance training can improve the postural stability and gait in elderly people, prevent the loss of muscle strength, and prevent the decline in cardiovascular circulation due to ageing. Recently, young people have been exercising less due to lifestyle changes. This has resulted in a poor muscle endurance and postural stability, which increases the risk of exercise injury or falls. In previous studies, mostly elderly subjects were used to show improvements in the postural stability and muscle strength. These studies did not clearly demonstrate the relationship between improved muscle strength and improved postural stability. Moreover, very few studies have used young subjects to simultaneously measure the centre of pressure (COP) and electromyography (EMG), and evaluate the correlation between postural stability and lower-limb muscles. Numerous noise sources may contaminate the EMG signal measurements and distort the signal, which can lead to interpretation errors of the EMG signal for investigating muscle activity. The variation in the COP measurements is relatively small. Therefore, entropy-based analyses for the application of COP may be enlarged if the relationship between the COP and EMG can be demonstrated.

The COP is the trajectory of the pressure centre acquired as a function of time as the body weight is transmitted to the ground when both feet are standing on the ground, which is important information for studying the postural stability and falls. There are some differences in the postural stability between young and elderly people. Hatton et al. [4] used conventional indicators of COP quantitation to examine patients who underwent cruciate-ligament reconstruction and found that there were no significant differences in the postural stability before and after surgery. Ozaki et al. [5] studied frail, elderly people and found that training with a newly-proposed, postural-stability-exercise-assist robot can improve postural stability; moreover, lower-limb muscle strength may improve faster using this technique compared to the conventional methods. Coelho et al. [6] studied the relationship between age and quality of standing balance in single and dual task conditions for the community-dwelling elderly. The results showed that the elderly were consistently associated with poor standing balance in single and dual task conditions. Bergamin et al. [7] studied the posture swaying in the various dual-task conditions of young people and old people. The results indicated that a simple verbal assignment was the secondary task which most influenced the postural balance and a dual-task condition seems to differently affect the balance variables, independently from age. The EMG measures weak potential changes produced during muscle contraction. The movement sequence of the agonist and antagonist muscles in a specific action can be revealed via EMG. Hägg et al. [8] highlighted that the EMG can be quantified to be a factor of the magnitude of force exerted and the level of fatigue. Muscles and bones play important roles in good postural stability. Müller et al. [9] studied the relationship between postural stability and lower-limb muscles in young and elderly people when standing using onset latency. The results demonstrated that the correlation coefficients between the tibialis anterior muscle and the anteroposterior direction in young and elderly people were 0.667 and 0.482, respectively. The correlation coefficients between the anteroposterior direction and the soleus and gastrocnemius muscles were less than 0.3. Borg et al. [10] studied the correlation between the postural stability and the gastrocnemius muscles in healthy people and patients with multiple sclerosis. He converted EMG into envelopes to calculate the correlation coefficient with anteroposterior COP. The results demonstrated that the correlation coefficient was 0.85, implying that gastrocnemius muscles are associated with the anteroposterior shaking of the body.

Previous studies utilised Fast Fourier Transform (FFT) for signal analysis. However, the FFT approach assumes a steady-state signal and linearity, which contradicts the signal characteristics of COP and EMG. Huang and Shen [11] proposed utilising the Hilbert–Huang Transform (HHT) for non-steady-state conditions and non-linear data, which can resolve the frequency range relevant for physical measurements. Amound et al. [12] adopted HHT for analysis and the results demonstrated that the frequency range from IMF1 to IMF5 can be used to identify the complex plane within a large area when the force plate vibrates. Andrade et al. [13] demonstrated that HHT can decrease the noise in EMG signals and that it performs better than wavelet transformation. Xie and Wang [14] calculated the mean EMG frequency during muscle fatigue, indicating that the HHT-estimated results have less variability and higher stability.

This study aims to see if the centre of pressure (COP) measurement on postural stability can be used to represent the electromyography (EMG) measurement of lower limb muscles activity data. If so, the cost-effective COP data measurement can be used to indicate the level of postural stability and lower limb muscles activity. Several experimental scenarios were designed for examining the effects of lower-limb muscles on postural stability in healthy, young people under dynamic and static exertion. An AMTI force plate and a NeXus-10 wireless physiological feedback system were used to collect the COP and EMG signals, respectively. HHT and Empirical Mode Decomposition (EMD) were used to identify the range of frequency characteristics under different states. Similarity indices and synchronization analyses were used to determine the correlation between lower-limb muscle strength and postural stability.

## 2. Methodology

The young people were used as experimental subjects, experiments were designed, and data analysis methods were proposed. Eight experimental scenarios were designed according to vision (open or closed eyes), posture (static or dynamic), and standing status (presence or absence of soft foam). An AMTI force plate and a NeXus-10 physiological feedback system were used to measure variations in postural stability and lower-limb muscles. Subsequently, EMD was utilised to decompose the COP and EMG signals into different IMF signals. Further, synchronization analyses were used to identify highly correlated COP and EMG data.

### 2.1. Test Subjects

The test subjects comprised of 12 healthy male and 12 healthy female undergraduate and graduate students with no mobility disorders. The eligibility criteria do not include the health or physical limitations that may affect the results of the study. The exclusion criteria included persons who had musculoskeletal or foot disorders or had undergone foot surgery or other invasive foot treatment procedures or mental illnesses. All participants provided their personal information before commencing the experiment. The average weight of males and females was 65 ± 7.53 kg and 45.7 ± 4.03 kg, respectively. Prior to the experiment, the subjects were informed about the experiment and experimental procedures in detail and the informed consent forms were signed.

### 2.2. Experimental Instrumentation

**Figure 1:**
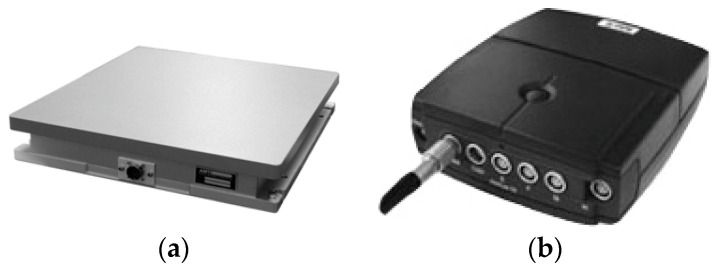
The experimental instruments. (**a**) AMTI force plate (AMTI^®^, Watertown, MA, USA) and (**b**) NeXus-10 physiological monitoring system (Mind Media^®^, BV, Herten, The Netherlands).

An AMTI force plate and a NeXus-10 physiological feedback system were used to collect the COP and EMG data, respectively (Figure 1a,b). The acquisition frequency of the force plate was 100 Hz. The acquisition frequency and measurement time of the wireless physiological feedback monitor were 1024 Hz and 60 s, respectively.

### 2.3. Experimental Procedures

**Figure 2:**
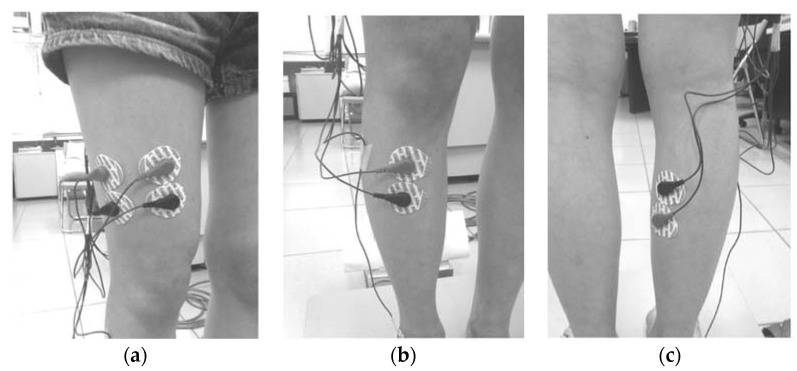
The electrode pads placed on different muscles: (**a**) rectus femoris and vastus lateralis; (**b**) tibialis anterior, and (**c**) gastrocnemius medialis.

**Figure 3:**
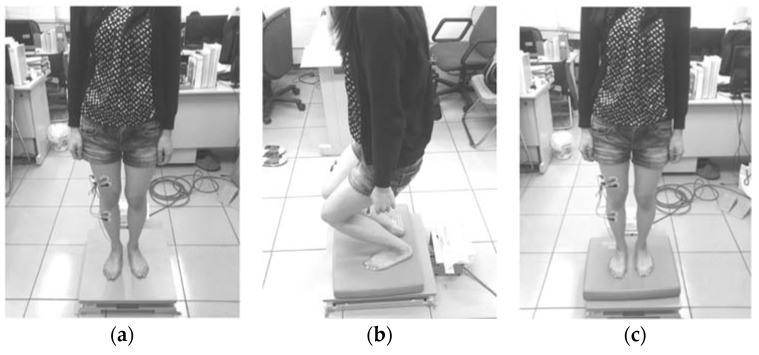
The experimental scenarios: (**a**) static experimental; (**b**) dynamic experimental; and (**c**) soft foam placed on the force plate.

A randomization method was used to arrange the experimental subjects. The rectus femoris (RF), vastus lateralis (VL), tibialis anterior (TA), and gastrocnemius medialis (GM) muscles were used as representatives of the lower-limb muscles. In the experimental procedure, each subject was swabbed with ethanol at the test muscle sites and electrode pads were attached (Figure 2). Subsequently, the maximum-voluntary-isometric contraction (MVC) was measured before the eight experimental scenarios were conducted in a random order with simultaneous COP and EMG measurements. In the study, a two-level experiment with three factors that includes vision (open or closed eyes), posture (static or dynamic) and standing status (presence or absence of soft foam) was designed with 2^3^ scenarios to collect data. The static experimental scenario refers to the experiments where the subjects were standing still (Figure 3a). The dynamic experimental scenario refers to the process whereby two instructions were randomly given to the subject: (1) squat to a half-squat position whilst keeping their heels on the ground and then (2) automatically rise up (Figure 3b). The soft foam was placed on the force plate and the subjects were asked to stand on it (Figure 3c). Each experiment was conducted for 60 s with a 3 min rest interval between experiments in order to recover and to avoid muscle fatigue [15].

**Figure 4:**
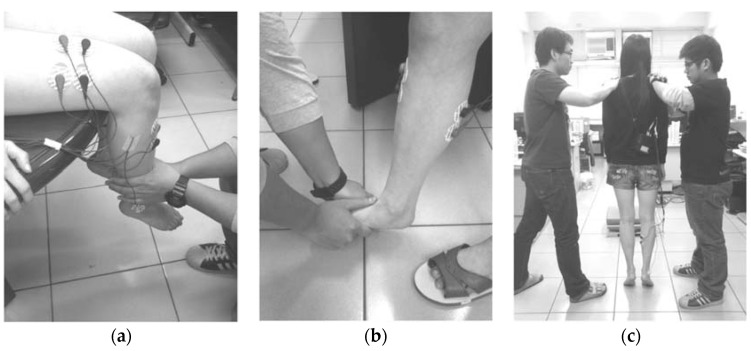
The measurement of maximum-voluntary-isometric contractions for (**a**) rectus femoris and vastus lateralis; (**b**) tibialis anterior; and (**c**) gastrocnemius medialis.

The MVC measurements were used as a standard reference to decrease muscle differences between different individuals. During the MVC measurements of the rectus femoris and vastus lateralis muscles, the subject adopted a sitting posture on the table, with the calf closely pressed against the edge of the table and both hands grabbing the table edge to fix the position of the upper trunk. The kneecap extends to resist the external force from the ankle for 5 s continuously (Figure 4a). During the MVC measurements of tibialis anterior muscles, the subject adopted a single-leg standing position with both knees fully extended. The maximum dorsiflexion force was continuously exerted on the external force for 5 s using one leg as a support (Figure 4b). During MVC measurements of the medial-gastrocnemius muscles, the subjects held a two-leg standing position with both feet exerting upward forces to act as a plantar flexion force, which continuously resists the external forces from the shoulder for 5 s (Figure 4c).

### 2.4. Analysis Algorithms

A two-stage procedure was used to analyse the COP and EMG signals. Firstly, the EMD was used to decompose the COP and EMG signals into several IMFs and two IMFs, and similar signal frequencies with COP and EMG were identified. During the second stage, the IMFs obtained from COP and EMG was used for correlation analyses to determine the relationship between postural stability and changes in the lower-limb muscles.

#### 2.4.1. Empirical Mode Decomposition

**Figure 5:**
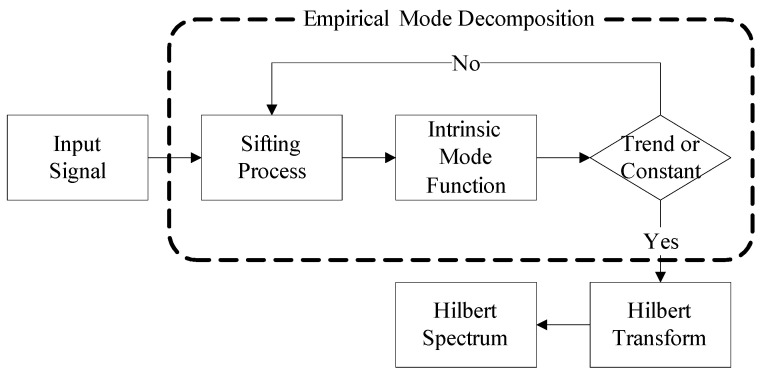
A flowchart of the Hilbert–Huang Transform analysis.

The greatest difference between HHT and wavelet transformations is the absence of basic functions. The HHT uses decomposition rules to replace basis functions and EMD is used to decompose signals into summations of IMFs. Subsequently, the Hilbert Transform method is used to obtain meaningful instantaneous frequencies. This is advantageous since it can analyse non-linear and non-steady-state signals and present the original physical characteristics of the signals. EMD and Hilbert Transform are the main procedures for HHT [11]. Figure 5 depicts the HHT analyses procedures.

EMD utilises the characteristic time scales in signals to define the vibrational mode and provide good vibrational mode analyses. A non-zero mean signal can also be used, and the decomposition procedure is termed sifting process [16,17,18], which is as follows:

Step 1. Identify the local maxima and local minima of the signal X(t) and use cubic splines to define the local maxima as the upper envelope and the local minima as the lower envelope. The mean of the upper and lower envelopes is taken as the mean envelope $m_{1}\left(t\right)$.

Step 2. The mean envelope $m_{1}\left(t\right)$ is subtracted from the signal X(t) to obtain the component $h_{1}\left(t\right)$.

$$h_{1}\left(t\right)=X\left(t\right)-m_{1}\left(t\right)$$

Step 3. Validate whether $h_{1}\left(t\right)$ fulfils the IMF conditions. The stopping criterion is that the difference between the number of extreme points and the number of cross-zero points must be less than or equal to one, and the mean envelope must be zero. If the stopping criterion is not fulfilled, return to Step 1 to use $h_{1}\left(t\right)$ as X(t) for the second sifting process to obtain $h_{11}\left(t\right)$.

$$h_{11}\left(t\right)=h_{1}\left(t\right)-m_{11}\left(t\right)$$

Step 4. The excessive screening will result in the loss of original physical characteristics from the results. Therefore, the sifting process must obey the convergence criteria to ensure that the physical characteristics of IMF are maintained. The convergence criteria are that the difference between the number of local extreme points and cross-zero points must be zero and the standard deviation should lie between 0.2 and 0.3. The formula is given as

$$SD=\overset{T}{\underset{t=0}{\sum }}{\left[\frac{\left(h_{1\left(k-1\right)}\left(t\right)-h_{1k}\left(t\right)\right)}{h_{1\left(k-1\right)}}\right]}^{2}$$

Step 5. After the *k* sifting processes, if the IMF stopping or convergence criteria are met, then component $h_{1k}\left(t\right)$ can be taken to be the first IMF and is represented by $c_{1}\left(t\right)$. $c_{1}\left(t\right)$ as the component with the shortest cycle in X(t).

$$h_{1k}\left(t\right)=h_{1\left(k-1\right)}\left(t\right)-m_{1k}\left(t\right)$$

$$c_{1}\left(t\right)=h_{1k}\left(t\right)$$

Step 6. $c_{1}\left(t\right)$ is subtracted from the signal X(t) to obtain the residual function $r_{1}\left(t\right)$. If $r_{1}\left(t\right)$ contains other components with longer cycles, then Steps 1–5 are repeated for $r_{1}\left(t\right)$ until *n* number of IMFs are selected.

$$X\left(t\right)-c_{1}\left(t\right)=r_{1}\left(t\right)$$

$$r_{1}\left(t\right)-c_{2}\left(t\right)=r_{2}\left(t\right)$$

⋮

$$r_{n-1}\left(t\right)-c_{n}\left(t\right)=r_{n}\left(t\right)$$

Step 7. When IMFs with physical characteristics cannot be obtained from the residual function $r_{n}\left(t\right)$, the sifting process is stopped. The last $r_{n}\left(t\right)$ obtained is used as the mean trend. The summation of every IMF and the mean trend can be used to reconstruct the initial signal X(t).

$$X\left(t\right)=\overset{n}{\underset{k=1}{\sum }}c_{k}\left(t\right)+r_{n}\left(t\right)$$

After COP and EMG signals were decomposed by EMD into several IMFs, Hilbert Transform was used to calculate the instantaneous frequency of every IMF, using the following formula:

$$Y\left(t\right)=\frac{1}{\pi }PV\overset{\infty }{\underset{-\infty }{\int }}\frac{X\left(\tau \right)}{t-\tau }d\tau$$

where Hilbert transform Y(t) can be obtained for any time series X(t) and *PV* represents Cauchy Principal Value.

Every X(t) that undergoes Hilbert transform becomes as follows

$$X\left(t\right)=\overset{n}{\underset{j=1}{\sum }}a_{j}\left(t\right)\exp\left(i\int w_{j}\left(t\right)dt\right)$$

#### 2.4.2. Similarity Index and Synchronization Analysis

This study uses the similarity index and synchronization analyses to investigate the correlation between the COP and EMG [19,20,21]. The similarity index is used to evaluate the similarity between two sets of signal fluctuation models and is obtained as follows:

Step 1. Decimal signals are converted to binary signals using the following formula:

$$I_{n}=\{\begin{array}{c} 0,\;if\;x_{n}\leq x_{n-1}\\ 1,\;if\;x_{n}>x_{n-1} \end{array}$$

**Figure 6:**
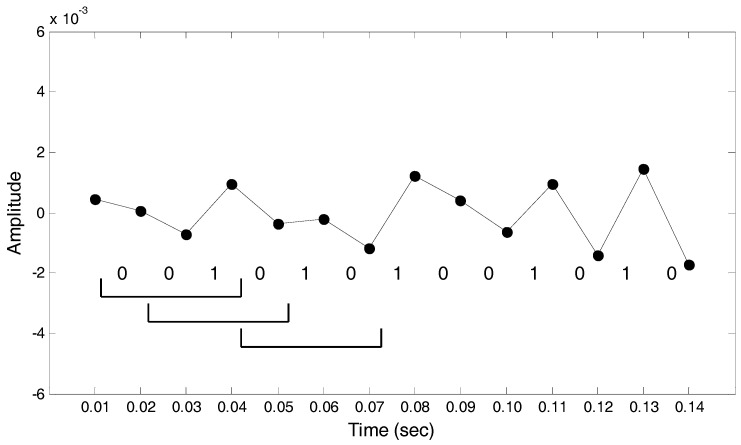
The original signal coding diagram for Steps 1 and 2.

In Figure 6, the first data point is greater than the second data point and the interval between the two is coded to equal zero. The same applies to the second interval. The third data point is smaller than the fourth data point, and the third interval is coded to equal one, and so on and so forth.

Step 2. The parameter *m* is set as the size of the moving window for use as the basis for the binary conversion to decimal. When *m* is set as 4, using the first moving window in Figure 6 as an example, the binary time-series of the window was 0010 and converted to decimal as 2. The binary time series of the second moving window was 0101 and converted to decimal as 5, and so on and so forth, to convert the binary time-series in the entire set into decimals.

**Figure 7:**
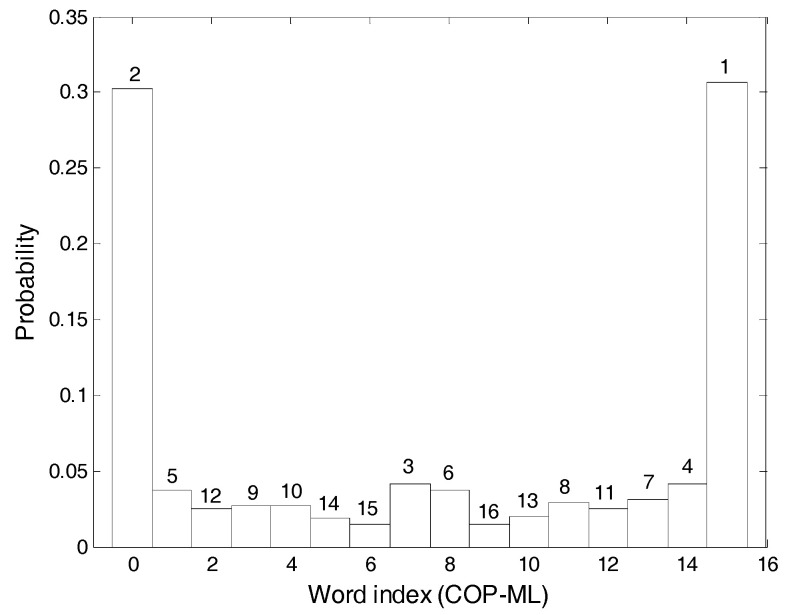
The example COP-ML (centre of pressure-medial lateral) signal results for Step 3.

Step 3. As *m* was set to be 4, the numbers 0 to 15 will appear in the binary to decimal conversion. The decimal time-series after conversion are tallied, the probabilities for the appearance of the numbers 0 to 15 are calculated, these are plotted as a histogram and the probabilities are ranked from largest to smallest. Figure 7 depicts the COP-ML (centre of pressure-medial lateral) signal results and the probability ranking of the numeral 0 was 2.

**Figure 8:**
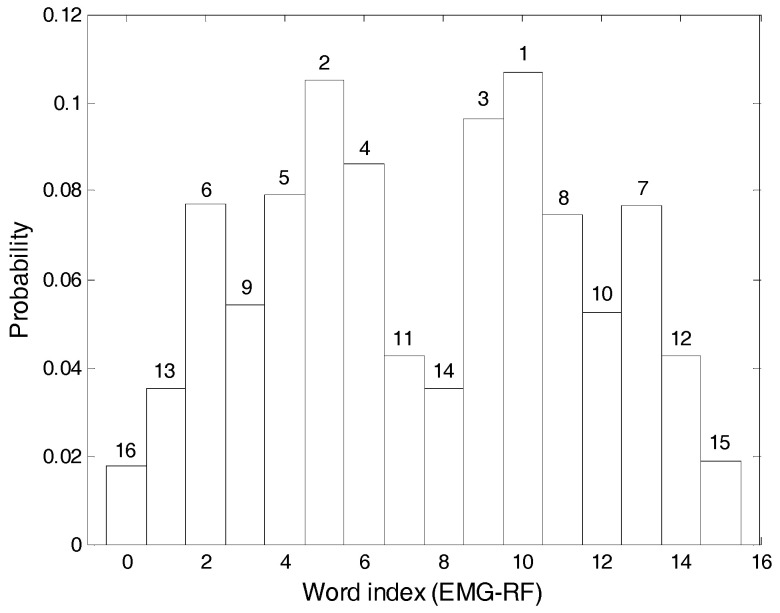
The example EMG-RF (electromyography-rectus femoris) signal results for Step 4.

Step 4. Steps 1–3 are repeated for another set of signals. Figure 8 depicts the results for the EMG-RF (electromyography-rectus femoris) muscles.

**Figure 9:**
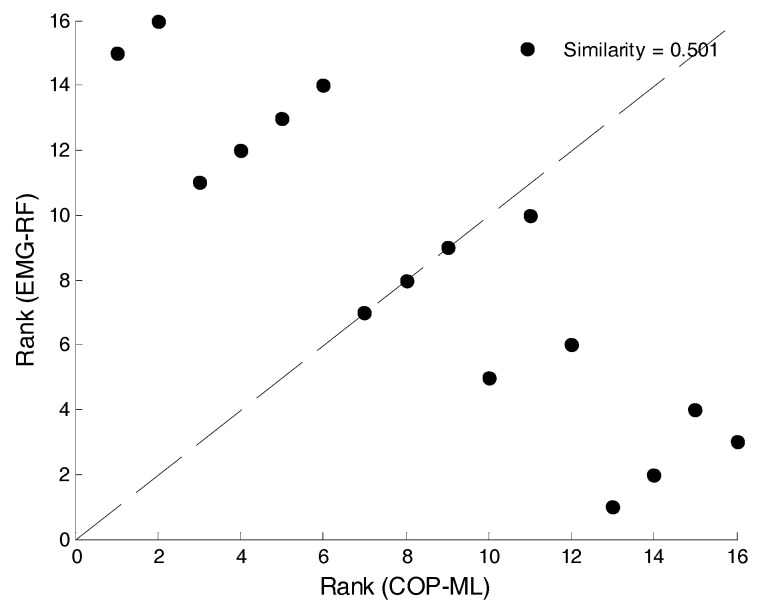
The scatterplot between Rank (EMG-RF) and Rank (COP-ML).

Step 5. Scatterplots are generated for the numeral ranking of two sets of signals that are to be compared. Figure 9 illustrates the results of COP-ML and EMG for the RF muscles. If the points in the scatterplot are more concentrated at the 45-degree diagonal dotted line, the similarity in the fluctuation methods of both sets of signals is high.

Step 6. The similarity index $D_{m}\left(\mathrm{COP},\mathrm{EMG}\right)$ of COP and EMG is

$$D_{m}\left(\mathrm{COP},\mathrm{EMG}\right)=1-\frac{\sum_{k=1}^{2^{m}}\left|R_{1}\left(w_{k}\right)-R_{2}\left(w_{k}\right)\right|F\left(w_{k}\right)}{2^{m}-1}$$

$$F\left(w_{k}\right)=\frac{\left(-p_{1}\left(w_{k}\right)\log\;p_{1}\left(w_{k}\right)-p_{2}\left(w_{k}\right)\log\;p_{2}\left(w_{k}\right)\right)}{\sum_{k=1}^{2^{m}}\left(-p_{1}\left(w_{k}\right)\log\;p_{1}\left(w_{k}\right)-p_{2}\left(w_{k}\right)\log\;p_{2}\left(w_{k}\right)\right)}$$

If $D_{m}\left(\mathrm{COP},\mathrm{EMG}\right)$ approaches one, the two sets of signals are similar. $R_{1}\left(w_{k}\right)$ represents the ranking of the *k*-th numeral of the first signal set and $p_{1}\left(w_{k}\right)$ is the probability of the *k*-th numeral appearing in the first signal set.

Synchronization indices are used to evaluate the degree of phase coordination between two sets of signals [22,23]. In this study, synchronization indices were used to evaluate the correlation of COP and EMG. The calculation of synchronization indices is based on the instantaneous phase and Shannon entropy and the process is as follows:

Step 1. The Y(t) obtained from the Hilbert Transform is expressed as complex numbers, Y(t)=a(t)+ib(t). The instantaneous phase θ(t) is calculated from the real part a(t) and the imaginary part b(t).

$$\theta \left(t\right)=\tan^{-1}\left(\frac{b\left(t\right)}{a\left(t\right)}\right)$$

Step 2. The instantaneous phases obtained from the two sets of signals are subtracted from each other to obtain the phase difference.

$$\mathrm{phase}\;\mathrm{difference}=\theta_{signal1}\left(t\right)-\theta_{signal2}\left(t\right)$$

Step 3. The absolute value of the phase difference is obtained to plot the histogram of *N* number of intervals.

phase difference(t)=|phase difference(t)|

$N=e^{0.626+0.4\times \ln(n-1)}$, *n* is the total length of the data.

Step 4. The Shannon entropy of the histogram is calculated.

$$\mathrm{Shannon}\;\mathrm{entropy}=\overset{N}{\underset{i=1}{\sum }}p\left(i\right)\times \ln\left(p\left(i\right)\right)$$

*p* is the probability of the occurrence for that interval.

Step 5. The synchronization indices are calculated.

$$\mathrm{Synchronization}\;\mathrm{index}=1-\frac{\mathrm{Shannon}\;\mathrm{entropy}}{\ln\left(N\right)}$$

## 3. Experimental Results

**Figure 10:**
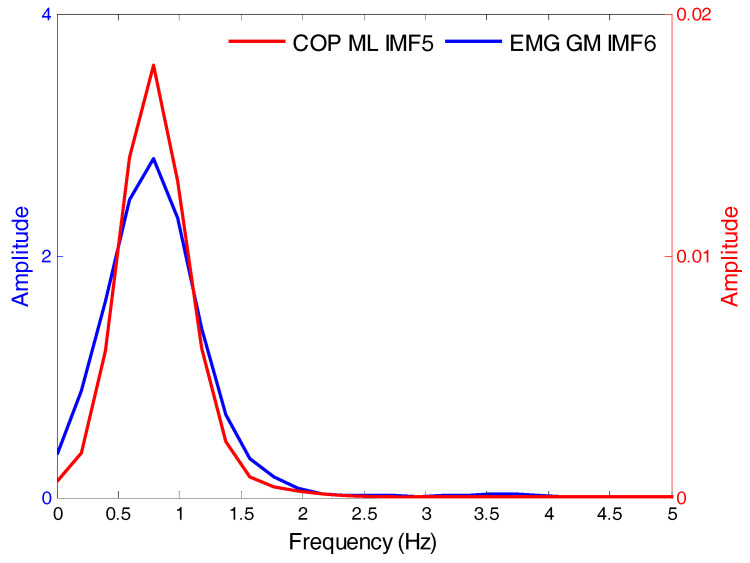
The COP and EMG frequency distribution for the experimental scenarios with eyes closed, standing still, and with soft foam. IMF: intrinsic-mode functions; EMG: Electromyography.

Firstly, the sampling was set to 100 Hz and experiments were designed to simultaneously measure the COP and EMG data. Subsequently, the EMD was used to decompose the COP and EMG signals into several IMFs and the average frequency of each IMF was calculated. The IMFs with similar COP and EMG signal-frequency-vibration modes were identified for synchronization analyses. Using data from closed eyes, standing still, and presence of soft foam as examples, Table 1 depicts the mean frequencies of each intrinsic-mode function in the left-right direction and anterior-posterior direction COP and the EMG of the RF, VL, TA, and GM. Statistical tests found that the IMF5 frequency of the COP signal and the IMF6 frequency of the EMG signal were not significantly different. Figure 10 depicts the frequency distribution of COP_IMF5_ and EMG_IMF6_. The frequency distribution of the two sets of signals were similar; the average frequency was 0.8 Hz with a range of 0–2 Hz.

**Figure 11:**
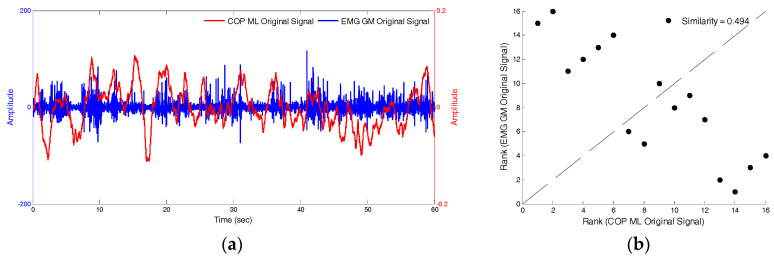
The raw-signal of the anterior-posterior COP and the EMG of MG muscles and the similarity index.

**Figure 12:**
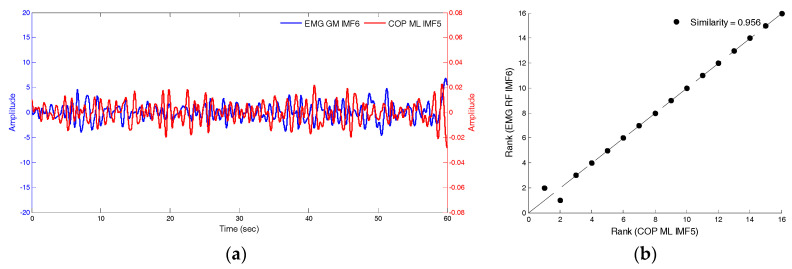
The signal functions of IMF5 of anterior-posterior COP and IMF6 of the EMG of MG muscles and the similarity index.

The similarity index was then used to compare the differences between the COP_IMF5_ and EMG_IMF6_ signals, and the original COP and EMG signals. Under the conditions of closed eyes, standing still, and presence of soft foam, Figure 11a,b depicts the raw-signal graph of the anterior-posterior COP the EMG of the medial-gastrocnemius muscles and the similarity index, which was 0.494. Figure 12a,b depicts that the signal graphs of IMF5 of anterior-posterior COP, the IMF6 of the EMG of medial-gastrocnemius muscles and the similarity index, which was 0.956. The fluctuation modes of the two sets of signals are extremely similar and the similarity index was higher relative to the raw-signal (0.462).

**Figure 13:**
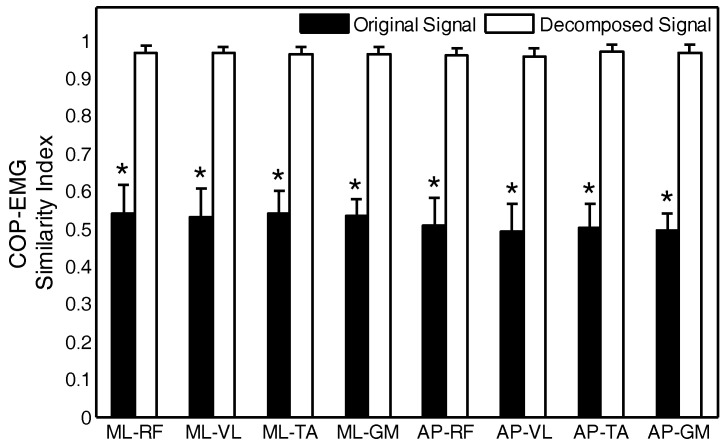
The comparative analyses of the similarity index between the original and decomposed signals. RF: rectus femoris; VL: vastus lateralis; TA: tibialis anterior; GM: gastrocnemius medialis; ML: medial-lateral; AP: anterior-posterior.

For the four muscles, the similarity between the raw signals and decomposed signals of the left-right direction and anterior-posterior direction, respectively, the COP and the EMG data were compared. The black histogram in Figure 13 depicts the similarity between the two sets of signals, and the white histogram depicts the similarity between the two sets of signals after decomposition. ML-RF is the similarity between the left-right COP and EMG of rectus femoris muscles. The * symbol indicates that there are statistically-significant differences between the two signals. The mean similarity index of the raw signals was 0.5–0.6. The similarity index between the IMF5 of the COP signal and the IMF6 of the EMG signal was increased to greater than 0.95. The COP_IMF5_ and EMG_IMF6_ obtained from the statistical tests and the similarity index was used to replace the raw signals for subsequent analysis.

## 4. Discussion and Conclusions

In this study, different scenarios were designed to simultaneously collect COP and lower-limb-muscle EMG data to investigate the correlation between these two sets of parameters. The Empirical Mode Decomposition was used to identify the intrinsic-mode functions (IMF) that can distinguish between COP and EMG at different states. Alickovic et al. [18] studied seizure onset detection and seizure onset prediction using EEG signals by the EMD, DWT (discrete wavelet transform), and WPD (wavelet packet decomposition). The results indicated that DWT or WPD is better than EMD for feature extraction. This study applied the empirical mode decomposition that has been demonstrated that COP may provide enough information on postural stability without EMG data. Therefore, there is no comparison with other methods.

The experimental analyses found that the IMFs that are similar for the COP and EMG signals are IMF5 and IMF6, respectively, and the average frequency was 0.8 Hz. The similarity index between the two was 0.95 to 1. This is higher than the similarity between the raw signals, which ranged from 0.5 to 0.6, and the difference was statistically significant. This is consistent with the results of Berg who highlighted that EMGs with frequencies less than 1 Hz were correlated with COP signals [23]. By analysing the synchronization indices, it was found that when postural stability is poor, EMG and COP were more synchronised with synchronisation indices approximately ranging from 0.010 to 0.015. With better postural stability, synchronization indices ranged from approximately 0.006 to 0.08 and both EMG and COP exhibited lower synchronization. Postural stability is relatively unstable when there is a greater synchronization between lower-limb muscle and postural stability. Conversely, when synchronization between lower-limb muscle and postural stability is low, postural stability is in a relatively stable state.

The results of this study and those from Manor et al. [24] are consistent. Manor et al. [24] remarked that there is an inverse correlation between the synchronization of respiratory flow and postural stability and postural stability. Conversely, lower synchronization leads to improved postural stability. Manor et al. [24] also noted that the synchronization index between respiratory flow and COP in normal people and stroke patients were approximately 0.11 and 0.25, respectively. This difference is greater than the synchronization index between EMG and COP, which ranged from 0.006 to 0.015. This is mainly because for muscles that affect postural stability, the same muscle does not continuously affect postural stability to maintain postural stability. Therefore, postural stability will possibly be affected by RF in the initial 10 s, and then the TA on the right leg will affect postural stability in the subsequent 10 s. This is different from breathing, which continuously affects postural stability. Therefore, the values from synchronization analyses in this study are less than or equal to respiratory flow and COP.

No fatigue was observed in the experimental data, which was used for joint analyses of the EMG spectrum and application, for any lower-limb muscle when subjects were standing for 1 min. In future research, increasing the duration that the subjects stand will be used to investigate how lower-limb-muscle fatigue affects the maintenance of postural stability. It is expected that the synchronization indices will increase when muscles are fatigued.

## Figures and Tables

**Table 1:** The statistical test results for each IMF of the left-right direction and anterior-posterior direction COP, and the EMG of the RF, VL, TA and GM.

	COP		EMG				p-Value			
	**ML**		**RF**	**VL**	**TA**	**GM**	**ML-RF**	**ML-VL**	**ML-TA**	**ML-GM**
IMF1	31.59 ± 0.25	IMF1	32.45 ± 1.00	32.73 ± 1.02	32.47 ± 0.77	32.75 ± 0.62	0.00	0.00	0.00	0.00
IMF2	15.02 ± 0.65	IMF2	15.69 ± 0.38	15.91 ± 0.44	15.75 ± 0.34	15.74 ± 0.32	0.00	0.00	0.00	0.00
IMF3	6.59 ± 0.59	IMF3	7.90 ± 0.32	7.99 ± 0.36	7.99 ± 0.26	8.08 ± 0.21	0.00	0.00	0.00	0.00
IMF4	2.28 ± 0.39	IMF5	1.82 ± 0.14	1.88 ± 0.16	1.88 ± 0.17	1.92 ± 0.23	0.00	0.00	0.00	0.00
IMF5	0.81 ± 0.12	IMF6	0.80 ± 0.12	0.80 ± 0.13	0.81 ± 0.16	0.88 ± 0.14	0.77	0.87	0.97	0.07
IMF6	0.38 ± 0.05	IMF7	0.34 ± 0.07	0.32 ± 0.07	0.34 ± 0.08	0.38 ± 0.09	0.02	0.00	0.05	0.96
	**AP**		**RF**	**VL**	**TA**	**GM**	**AP-RF**	**AP-VL**	**AP-TA**	**AP-GM**
IMF1	31.46 ± 0.31	IMF1	32.45 ± 1.00	32.73 ± 1.02	32.47 ± 0.77	32.75 ± 0.62	0.00	0.00	0.00	0.00
IMF2	15.24 ± 0.56	IMF2	15.69 ± 0.38	15.91 ± 0.44	15.75 ± 0.34	15.74 ± 0.32	0.00	0.00	0.00	0.00
IMF3	6.12 ± 1.09	IMF3	7.90 ± 0.32	7.99 ± 0.36	7.99 ± 0.26	8.08 ± 0.21	0.00	0.00	0.00	0.00
IMF4	2.26 ± 0.33	IMF5	1.82 ± 0.14	1.88 ± 0.16	1.88 ± 0.17	1.92 ± 0.23	0.00	0.00	0.00	0.00
IMF5	0.87 ± 0.14	IMF6	0.80 ± 0.12	0.80 ± 0.13	0.81 ± 0.16	0.88 ± 0.14	0.07	0.09	0.19	0.75
IMF6	0.40 ± 0.04	IMF7	0.34 ± 0.07	0.32 ± 0.07	0.34 ± 0.08	0.38 ± 0.09	0.00	0.00	0.00	0.29

Note: The bold text indicates statistical significance (*p*-value < 0.05), where ML-RF indicates ML and RF were used to perform statistical hypothesis tests. IMF: intrinsic-mode functions; COP: centre of pressure; EMG: electromyography; RF: rectus femoris; VL: vastus lateralis; TA: tibialis anterior; GM: gastrocnemius medialis; ML: medial-lateral; AP: anterior-posterior.
